# Development of Hydrosalpinx After Prior Vaginal Hysterectomy and Bilateral Salpingectomy

**DOI:** 10.7759/cureus.52573

**Published:** 2024-01-19

**Authors:** Jennifer M Lai, Megan Masten, Amy Markese

**Affiliations:** 1 Department of Obstetrics and Gynecology, University of Colorado Anschutz Medical Campus, Aurora, USA

**Keywords:** laparoscopic hysterectomy, total vaginal hysterectomy, salpingectomy, pelvic pain, hydrosalpinx

## Abstract

Hydrosalpinx is defined as the obstruction and fluid distension of the fallopian tube. It is most often seen in the setting of pelvic inflammatory disease, but preserved fallopian tubes or tubal segments after hysterectomy can also develop hydrosalpinx. This case report highlights an instance of painful hydrosalpinx after vaginal hysterectomy and advocates for the complete removal of fallopian tubes as the standard of care at the time of hysterectomy of any route. In this case, a 40-year-old female, G4P3104, with a history of vaginal hysterectomy and prophylactic bilateral salpingectomy for abnormal uterine bleeding and symptomatic uterine leiomyoma two years prior, presented with one month of left lower quadrant pain. She was found to have an anechoic, tubular structure adjacent to the left ovary on transvaginal ultrasound. At the time of diagnostic laparoscopy, a 10x4 centimeter (cm) dilated hydrosalpinx was found and removed. Pathology confirmed the hydrosalpinx, and the patient's pain resolved after the surgery. Given our findings of painful hydrosalpinx following incomplete bilateral salpingectomy at the time of vaginal hysterectomy, attempts at the removal of the entire fallopian tube including the fimbriae are strongly recommended to prevent the morbidity of repeated surgery.

## Introduction

Hydrosalpinx is a condition in which the fallopian tube becomes obstructed and distended with fluid accumulation. It is commonly caused by pelvic inflammatory disease. However, other less common pathological causes include endometriosis, malignancy, and adhesions from prior pelvic surgeries including prior tubal procedures. While this tubal disorder can be asymptomatic in some, it can lead to pain, torsion, ectopic pregnancy, and tubal factor infertility in others.

In the last two decades, there has been a shift toward routinely performing bilateral salpingectomy at the time of hysterectomy [1,2]. Initial concerns for long-term effects on ovarian function have been disproven [3]. Recent studies show increasing evidence that serous tubal intraepithelial carcinoma is the precursor for high-grade serous ovarian cancer [4,5]. It has thus become more accepted that removing the fallopian tubes may help to prevent ovarian, peritoneal, and tubal cancer.

Various methods have been described for the removal of fallopian tubes at the time of vaginal hysterectomy. Given that visualization may be somewhat limited, it is often more technically challenging to remove the entirety of the fallopian tube during a vaginal hysterectomy as compared to a laparoscopic or open abdominal procedure. There are limited case reports describing the formation of hydrosalpinx following a vaginal hysterectomy. This case report aims to demonstrate the importance of resecting the entire fallopian tube at the time of vaginal hysterectomy to prevent the complication of painful hydrosalpinx. 

## Case presentation

A 40-year-old female, G4P3104, had a total vaginal hysterectomy with concomitant bilateral salpingectomy and McCall culdoplasty performed two years prior for abnormal uterine bleeding. Before her hysterectomy, she was found to have a large submucosal fibroid measuring up to 4.8 cm on transvaginal ultrasound. Her additional workup was negative for cervical intraepithelial malignancy and endometrial hyperplasia or malignancy. Given that she failed to achieve resolution with multiple medical management options, including combined oral contraceptives, Provera (Pfizer, New York, New York, United States) and Aygestin (Amneal Pharmaceuticals, Bridgewater, New Jersey, United States), the patient was counseled at the time to undergo hysteroscopic polypectomy with intrauterine device (IUD) placement versus hysterectomy. The patient strongly desired definitive surgical management. Per chart review, the patient's uterus size and mobility made her a good candidate for vaginal hysterectomy. The surgical histopathology report confirmed multiple uterine fibroids, one fallopian tube measuring 3 cm in length × 0.4 cm in diameter, and a second fragmented fallopian tube measuring 2.3 cm in aggregates × 0.3 cm in maximal diameter. No adenomyosis, endometriosis, or other pathologies were described in the pathology or operative note.

**Figure 1:**
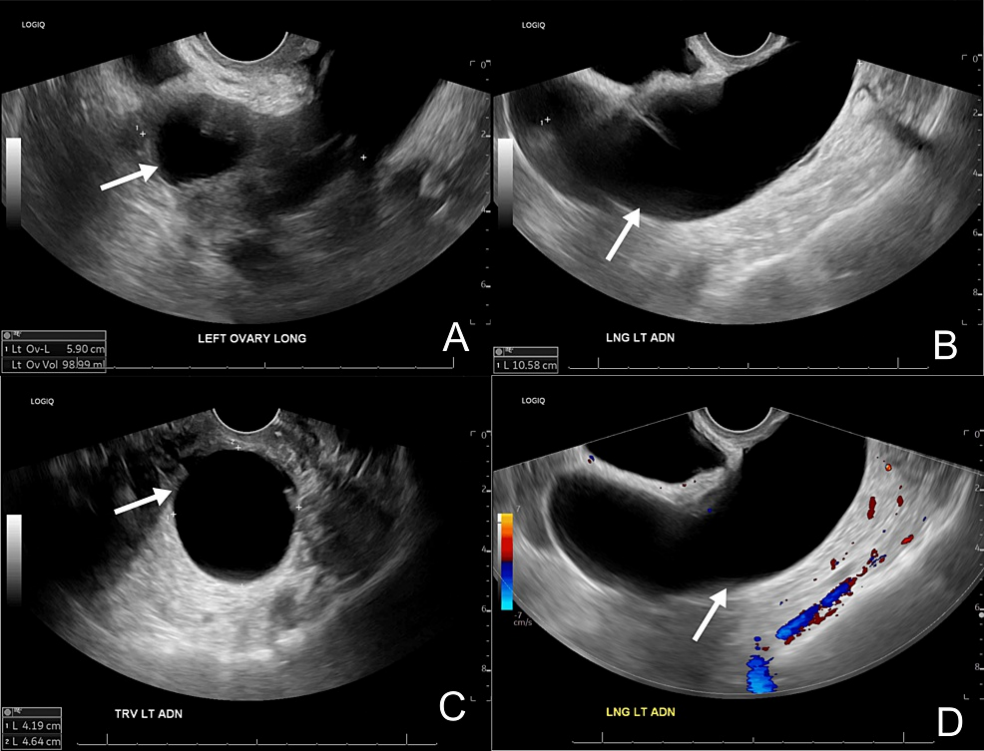
Transvaginal ultrasound Transvaginal ultrasound shows (A) the left ovary in the long axis that is attached to (B) a tubular-shaped, anechoic cyst. (C) Transverse view of the tubular-shaped cyst. (D) Doppler study shows an avascular tubular mass.

Two years later, the patient was referred to the Obstetrics and Gynecology clinic for left lower quadrant pain. The patient reported one month of left pelvic pain without any known injuries or relevant history of bowel or bladder symptoms. She described the pain as persistent, progressing from more moderate to severe pain that limited her daily function. She endorsed associated nausea and denied vaginal bleeding, discharge, fever, chills, or any issues with urination or bowel movements. Physical exam was significant for left lower quadrant pain and guarding, as well as a surgically absent cervix and uterus without any palpable adnexal mass on the bimanual exam. A transvaginal pelvic ultrasound with a Doppler study was obtained during her workup which revealed a 5.9 cm left ovary as shown in Figure 1A. The left ovary was adjacent to a 10.6x4.2x4.6 cm anechoic, avascular, tubular structure as seen in Figures 1B-1D. The right ovary and vaginal cuff were normal appearing. 

At this point, the differential diagnosis included a hydrosalpinx in a tubal remnant versus a simple ovarian cyst. Although ovarian cancer was considered, this was less likely with the absence of any malignant features, including septations, papillary projections, heterogenous echogenicity, solid components, or central vascularization. CA-125 was deferred as she was at low risk for ovarian cancer. The patient was offered surgical management or magnetic resonance imaging (MRI) for a definitive diagnosis. Due to the severity of the pain, she requested surgical management as soon as possible. During the preoperative visit one month later, the patient reported persistent left pelvic pain with worsening nausea and now mild vomiting, early satiety, and decreased appetite. She consented to an exam under anesthesia, laparoscopic bilateral salpingectomy to remove tubal remnants, possible ovarian cystectomy, and possible oophorectomy. Risks and benefits were discussed, including pain, bleeding, infection, damage to surrounding structures, and the possibility of not improving her pain.

**Figure 2:**
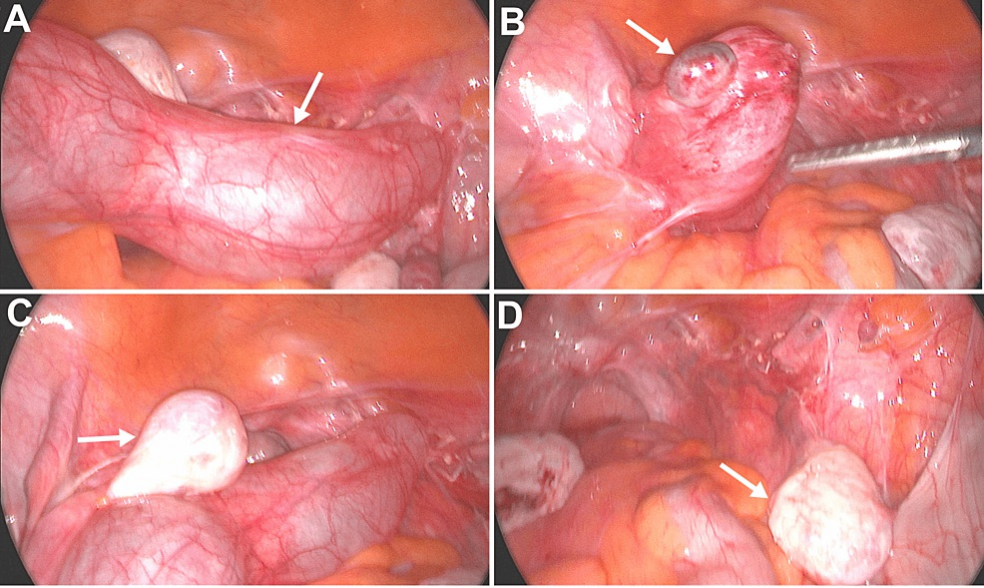
Intraoperative views during laparoscopy (A) A tubular-shaped cystic mass with (B) a chocolate-filled cyst at the proximal end is attached to (C) the normal-appearing left ovary. (D) The right ovary appears normal.

During her diagnostic laparoscopy, a left-sided 10x4 cm hydrosalpinx was noted with a dark cystic area near the proximal end as shown in Figures 2A-2B. There were a normal-appearing left ovary, a normal-appearing small proximal remnant of the right fallopian tube, and a normal-appearing right ovary as can be seen in Figures 2C-2D. The right and left ovaries were similar in size, suggesting the size of the left ovary measured on ultrasound was likely overestimated to include part of the hydrosalpinx. The hydrosalpinx was completely resected with bipolar cautery in the usual manner, and the right fallopian tubal remnant was removed as well. The patient tolerated the procedure well and was discharged home later that day. Histopathology report confirmed a fallopian tube with hydrosalpinx and a small endometriotic cyst. At her postoperative visit, the patient was doing well with resolved pain.

## Discussion

Hydrosalpinx is an abnormal collection of fluid within the fallopian tube. Most studies look at hydrosalpinx as a sequela of pelvic infections. With few studies discussing the iatrogenic causes of hydrosalpinx, this case presentation highlights the development of hydrosalpinx as a result of an incidentally retained tubal remnant despite undergoing a total vaginal hysterectomy with prophylactic bilateral salpingectomy. 

The initial concern for prophylactic bilateral salpingectomy was impairment of the ovarian blood supply given their close anatomic location and subsequent reduced ovarian function. However, many studies have refuted this argument, including a retrospective study showing no significant difference in ovarian hormone secretion in patients before and after their hysterectomy with concurrent total bilateral salpingectomy [3]. A randomized study also demonstrated no significant difference between ovarian blood supply on Doppler studies preoperatively and at one and six months after hysterectomy with and without bilateral salpingectomy [6].

Total bilateral salpingectomy has been suggested to be performed routinely with hysterectomy and in place of partial salpingectomy or other methods of tubal ligation by many studies to reduce the risk of benign adnexal pathologies and surgical complications requiring repeat surgical intervention. In a 2006 prospective cohort study, a 7.8% lifetime risk of subsequent surgery was found in women with a history of tubal ligation [7]. A retrospective cohort study in 2014 similarly compared that in premenopausal women, standard hysterectomy without prophylactic bilateral salpingectomy was found to have significantly twice the incidence of benign adnexal pathologies, including ovarian cysts, hydrosalpinx, pyosalpinx, and adnexitis, and significantly triple the rate of surgical re-intervention related to initial hysterectomies compared to the group with concurrent removal of the tubes [8]. These studies also show that patients undergoing only hysterectomy have an increased risk of surgical re-intervention due to infectious adnexal pathology compared to those undergoing hysterectomy with concurrent bilateral salpingectomy [7,8]. 

In addition to reducing benign adnexal pathologies, bilateral salpingectomy can reduce risks of ovarian, tubal, and peritoneal cancer. Fallopian tubes are found to serve as a source for high-grade serous ovarian carcinoma and a conduit for irritants, inflammation, and retrograde menstruation to potentially develop endometroid and clear cell carcinoma [9,10]. Given the lack of screenings for ovarian cancer and its poor prognosis, many studies support the additional step of removing fallopian tubes at the time of hysterectomy for benign pathologies in premenopausal women as a standard of care to reduce ovarian cancer risk [11,12]. Studies have also found that including bilateral salpingectomy during a hysterectomy has not increased intraoperative complications, hospitalization stay, or emergency readmission compared to hysterectomy alone [13]. 

Although removing bilateral tubes during hysterectomy has not been shown to have worse intraoperative complications, salpingectomy, especially from a vaginal approach, needs to be performed with caution to prevent the development of hydrosalpinx after surgery. It is crucial to have clear dissection with maximum visualization of the fallopian tube in order to remove the entire length of the fallopian tube from the isthmus to the fimbriae. There are multiple approaches described in recent studies to make salpingectomy easier and safer during vaginal hysterectomy, including the round ligament technique, posterior rotation of the uterus, and performing laparoscopically assisted vaginal hysterectomy (LAVH) instead of total vaginal hysterectomy [14-16]. An article in 2018 describes a technique that includes posterior rotation of the uterus during vaginal hysterectomy to remove tubes with reported success in all attempts in over 60 cases [15]. A recent study of a cohort with pelvic organ prolapse found a 0% failure rate to perform intended salpingo-oophorectomy in LAVH compared to a 36% failure rate in total vaginal hysterectomy [16]. LAVH was also shown to have a shorter operation time, earlier hospital discharge, and lower Clavien-Dindo classification of surgical complications than total vaginal hysterectomy, suggesting that LAVH may be superior to vaginal hysterectomies at removing adnexal structures [16].

Overall, it is noteworthy to consider hydrosalpinx in the differential diagnosis for any female with pelvic pain and a history of hysterectomy with salpingectomy, isolated salpingectomy, or tubal sterilization. This diagnosis can be supported by ultrasound and MRI. On ultrasound, a tubular, avascular, anechoic structure with a waist sign, or diametrically opposed indentation of the walls, is pathognomonic for hydrosalpinx [17]. This contrasts an ovoid-shaped structure which is more consistent with an ovarian cyst. On MRI, a fluid-filled C- or S-shaped tubular structure lateral to the uterus is consistent with hydrosalpinx [18]. 

## Conclusions

This case report advocates for prophylactic bilateral salpingectomy to be performed at the time of hysterectomy if feasible surgically for many clinical benefits and risk reduction of both benign and malignant adnexal pathologies. Due to the technical challenges of salpingectomy from a vaginal approach, maximizing visibility when removing the entire length of the fallopian tubes may be imperative to prevent the formation of hydrosalpinx and repeat surgical interventions. Several approaches are described here to improve the outcomes of salpingectomy from a vaginal route. However, additional studies and expert opinions are needed to provide a consensus on the best approach for achieving complete salpingectomy during vaginal hysterectomy. 
